# Discordant Radiologic and Biochemical Response With Inflammatory Activity in Metastatic Prostate Cancer: A Case Report

**DOI:** 10.7759/cureus.109495

**Published:** 2026-05-23

**Authors:** Ivan Bivolarski, Martin Dimitrov, Tsvetelina Yordanova

**Affiliations:** 1 Medical Oncology, Chronotherapy and Cancer Research Unit, Integrated Oncology Centre Burgas, Burgas, BGR; 2 Visceral Surgery, Klinik für Allgemeine, Gefäß- und Viszerale Chirurgie, Kreiskrankenhaus Greiz, Greiz, DEU; 3 Nuclear Medicine, Metabolic Therapy and Radiotherapy, Medical University “Prof. Dr. Paraskev Stoyanov”, Varna, BGR

**Keywords:** biomarkers, castration-resistant prostate cancer, discordant response, ga68 psma pet/ct, metastatic castration resistant prostate cancer, neutrophil-to-lymphocyte ratio (nlr), prostate-specific antigen (psa), spect/ct, tumor heterogeneity

## Abstract

Discordance between imaging findings and biochemical markers represents a significant diagnostic and therapeutic challenge in metastatic prostate cancer. We report the case of a 72-year-old male with high-risk prostate adenocarcinoma (Gleason 4+5=9) and extensive bone metastases, who demonstrated partial radiologic and metabolic response on prostate-specific membrane antigen PET/CT and single-photon emission CT/CT imaging following systemic therapy while simultaneously exhibiting rising prostate-specific antigen levels and elevated systemic inflammatory markers.

Despite imaging-defined response, CT revealed persistent mixed osteoblastic and osteolytic bone lesions, suggesting underlying biological heterogeneity and asynchronous tumor behavior. This discordant pattern indicates that radiologic improvement may not fully reflect ongoing systemic disease activity.

This case supports the hypothesis that systemic inflammatory activity, in conjunction with biochemical progression, may reflect early tumor-host interaction and emerging treatment resistance, potentially preceding overt radiologic progression.

These findings underscore the need for integrated, multimodal monitoring strategies combining imaging, biochemical, and inflammatory markers to better capture dynamic disease activity and enable earlier detection of treatment resistance in metastatic prostate cancer.

## Introduction

Metastatic prostate cancer is commonly monitored using a combination of imaging modalities and biochemical markers, particularly prostate-specific antigen (PSA). PSA remains a widely used surrogate marker of disease burden and treatment response, although its interpretation may become challenging in the setting of tumor heterogeneity, treatment-related fluctuations, and evolving therapeutic strategies [1]. In parallel, advances in imaging, including prostate-specific membrane antigen (PSMA) PET/CT and hybrid techniques such as single-photon emission CT (SPECT)/CT, have substantially improved the detection and characterization of metastatic disease, particularly skeletal involvement [2].

Despite these advances, discrepancies between radiologic findings and biochemical progression are increasingly recognized in clinical practice. In some patients, imaging may demonstrate stable disease or partial radiologic response, while PSA levels continue to rise, suggesting ongoing biological tumor activity. Such discordance presents a diagnostic and therapeutic challenge, raising questions regarding the true extent of disease activity and the limitations of relying on a single monitoring modality [3].

Metastatic prostate cancer is increasingly understood as a biologically dynamic and heterogeneous disease composed of multiple tumor subclones with variable sensitivity to systemic therapy [4]. Consequently, different metastatic compartments may respond asynchronously, potentially leading to inconsistencies between imaging findings, biochemical markers, and clinical evolution.

In addition to PSA kinetics and imaging findings, systemic inflammatory markers have emerged as potential adjunctive indicators of disease activity and prognosis. Among these, the neutrophil-to-lymphocyte ratio (NLR) has been investigated as a readily available biomarker associated with systemic inflammation, tumor-host interaction, and adverse oncologic outcomes in advanced prostate cancer [5]. However, the clinical interpretation of inflammatory biomarkers remains complex, and their role in monitoring treatment response or early progression is not yet fully established.

Although discordant biochemical and radiologic responses have been increasingly described, reports integrating longitudinal imaging findings, PSA dynamics, and inflammatory markers remain limited. Such observations may provide additional insight into the temporal complexity of metastatic disease evolution and treatment resistance.

Recognizing and appropriately interpreting these discordant patterns may contribute to a more comprehensive assessment of disease activity in metastatic prostate cancer. In this context, integrated evaluation of imaging, biochemical, and inflammatory parameters may support more individualized clinical monitoring strategies.

This case has not been previously presented or published in any form, including conference abstracts or scientific meetings.

## Case presentation

A 72-year-old male patient presented in October 2021 with progressive lower back pain, fatigue, and reduced physical activity. Clinical evaluation revealed impaired performance status (ECOG X) and findings suggestive of advanced metastatic disease. Prostate biopsy confirmed high-risk prostate adenocarcinoma with a Gleason score of 4+5=9. Initial PSA level exceeded 100 ng/mL (reference range: <4 ng/mL).

Baseline imaging evaluation demonstrated extensive skeletal metastatic involvement. At the time of initial assessment, imaging selection was influenced by local availability and institutional diagnostic workflow. An ¹⁸F-FDG PET/CT performed on December 2, 2022 demonstrated limited metabolic activity and did not clearly delineate the full extent of skeletal disease involvement. This observation is consistent with the recognized limited sensitivity of FDG-based imaging in prostate cancer, particularly in predominantly osteoblastic metastases (Figure 1).

**Figure 1:**
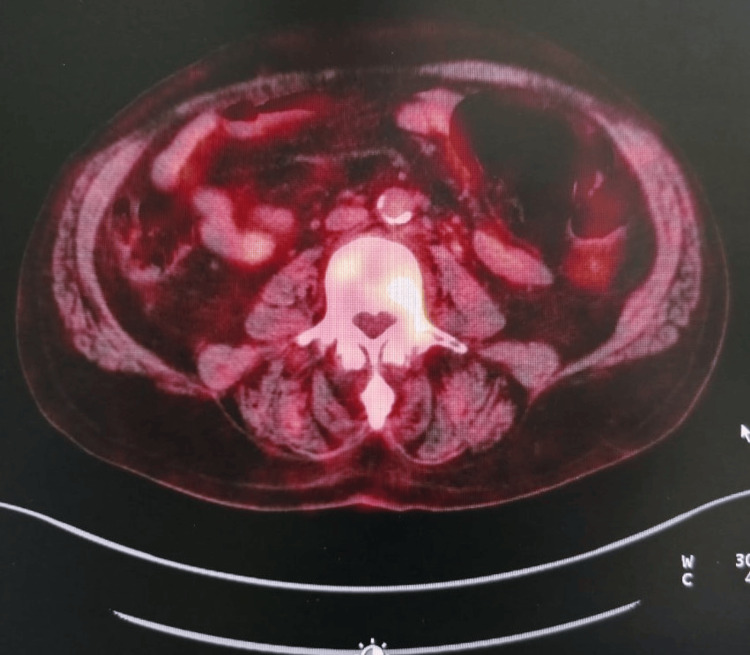
Axial FDG PET/CT (December 2, 2022) with limited sensitivity for prostate cancer metastases FDG-based imaging demonstrates relatively low tracer uptake, highlighting the known limitations of FDG PET/CT in detecting prostate cancer metastases, particularly in predominantly osteoblastic disease.

Following diagnosis, the patient was initiated on androgen deprivation therapy (ADT) with leuprorelin depot in combination with a bone-modifying agent (denosumab). Due to symptomatic skeletal metastatic disease, palliative radiotherapy was administered to the thoracic spine and pelvic region, resulting in partial symptomatic improvement.

A baseline ⁶⁸Ga-PSMA PET/CT performed in 2023 demonstrated extensive skeletal metastatic involvement, predominantly affecting the axial skeleton (Figure 2). Compared with the previously performed ¹⁸F-FDG PET/CT, PSMA-based imaging provided substantially improved visualization of metastatic bone disease burden.

**Figure 2:**
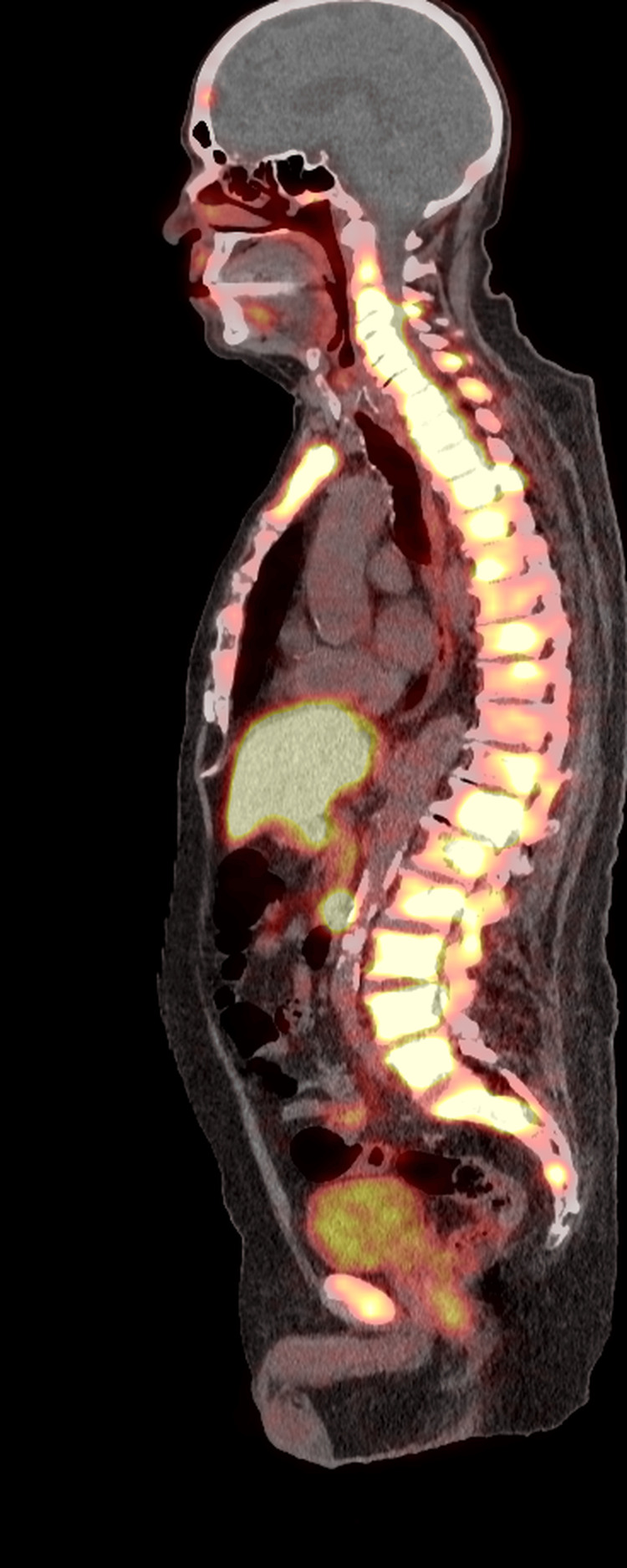
Sagittal PSMA PET/CT demonstrating axial skeletal metastases. Fused PSMA PET/CT image (2023) showing diffuse radiotracer uptake along the axial skeleton, consistent with extensive bone metastases. Physiological uptake is noted in the liver and urinary bladder. Focal pelvic uptake corresponds to the primary prostate tumor. PSMA, prostate-specific membrane antigen.

In January 2023, systemic therapy with enzalutamide was initiated as part of the treatment strategy for metastatic hormone-sensitive prostate cancer.

Longitudinal hematologic follow-up demonstrated temporal variation in immune cell distribution during the course of treatment. Between January 4, 2022 and January 3, 2023, granulocyte percentage gradually declined from 75.2% to 56.7% (reference range: 40%-75%), while lymphocyte percentage remained within the upper-normal range with moderate variability (reference range: 20%-45%).

On November 11, 2022, the PSA level was 278 ng/mL (reference range: <4 ng/mL), while the lymphocyte percentage measured 29.1%. Over the following months, PSA increased substantially, reaching 924 ng/mL on March 1, 2023, prompting initiation of systemic treatment with enzalutamide.

Following treatment initiation, biochemical response was observed, accompanied by moderate changes in hematologic inflammatory parameters, including a relative increase in granulocyte percentage and a reduction in lymphocyte proportion.

A marked PSA decline subsequently occurred, with levels decreasing to 4.23 ng/mL on March 28, 2023. Biochemical disease control was maintained over an extended period, with PSA levels of 3.251 ng/mL on July 23, 2024, and 5.128 ng/mL on November 18, 2024 (reference range: <4 ng/mL), consistent with a prolonged biochemical response to therapy (Figure 3).

**Figure 3:**
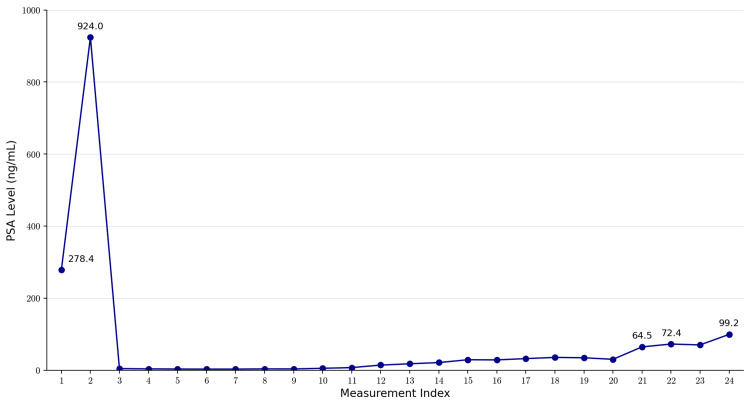
Temporal dynamics of prostate-specific antigen (PSA) levels across sequential measurements, demonstrating an initial peak, marked post-treatment decline, and subsequent gradual biochemical progression.

In June 2025, biochemical progression was documented, with PSA increasing to 17 ng/mL (reference range: <4 ng/mL), prompting initiation of first-line chemotherapy with docetaxel, while ADT was continued. The patient completed three cycles by August 2025. During this period, ADT was transitioned from leuprorelin depot to goserelin as part of ongoing hormonal management.

During 2025, gradual changes in hematologic inflammatory parameters were observed in parallel with biochemical progression. Granulocyte percentage increased from 69.9% on August 1, 2025 (reference range: 40%-75%) and reached its highest recorded value on October 3, 2025. Over the same interval, PSA levels increased from 32.179 ng/mL on September 15, 2025 to 35.17 ng/mL on October 3, 2025 (reference range: <4 ng/mL).

Although these temporal changes may reflect evolving systemic disease activity, the observed inflammatory alterations remained modest and should be interpreted cautiously within the broader clinical and radiologic context (Figure 4).

**Figure 4:**
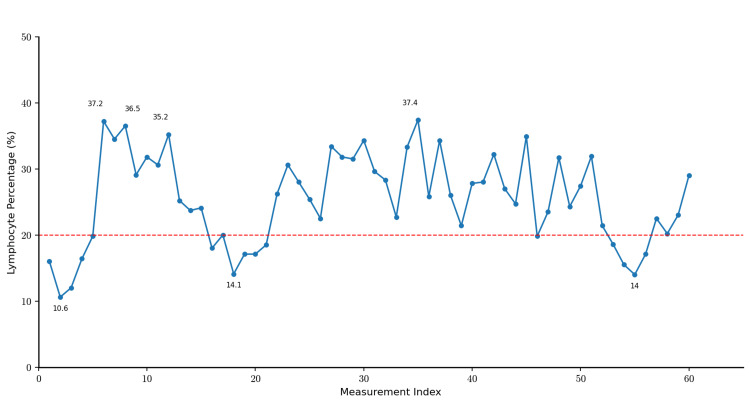
Temporal variation in lymphocyte percentage across sequential measurements, with a reference threshold at 20%

Following the initial post-treatment phase, longitudinal granulocyte measurements demonstrated variable fluctuations over time, characterized by alternating periods of increase and decrease. These temporal variations formed a visually apparent oscillatory pattern on serial assessment, although the clinical significance of this observation remains uncertain (Figure 5).

**Figure 5:**
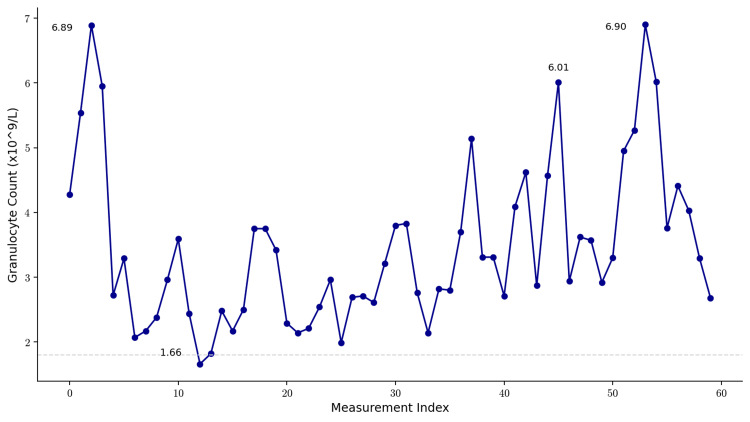
Temporal variation in granulocyte count across sequential measurements, demonstrating fluctuations around the lower reference threshold and intermittent peak elevations.

A prior ⁶⁸Ga-PSMA PET/CT performed in February 2025 demonstrated extensive osteoblastic metastatic involvement of the axial and appendicular skeleton, with an overall impression of partial response to systemic therapy. No visceral or nodal metastatic disease was identified (Figure 6).

**Figure 6:**
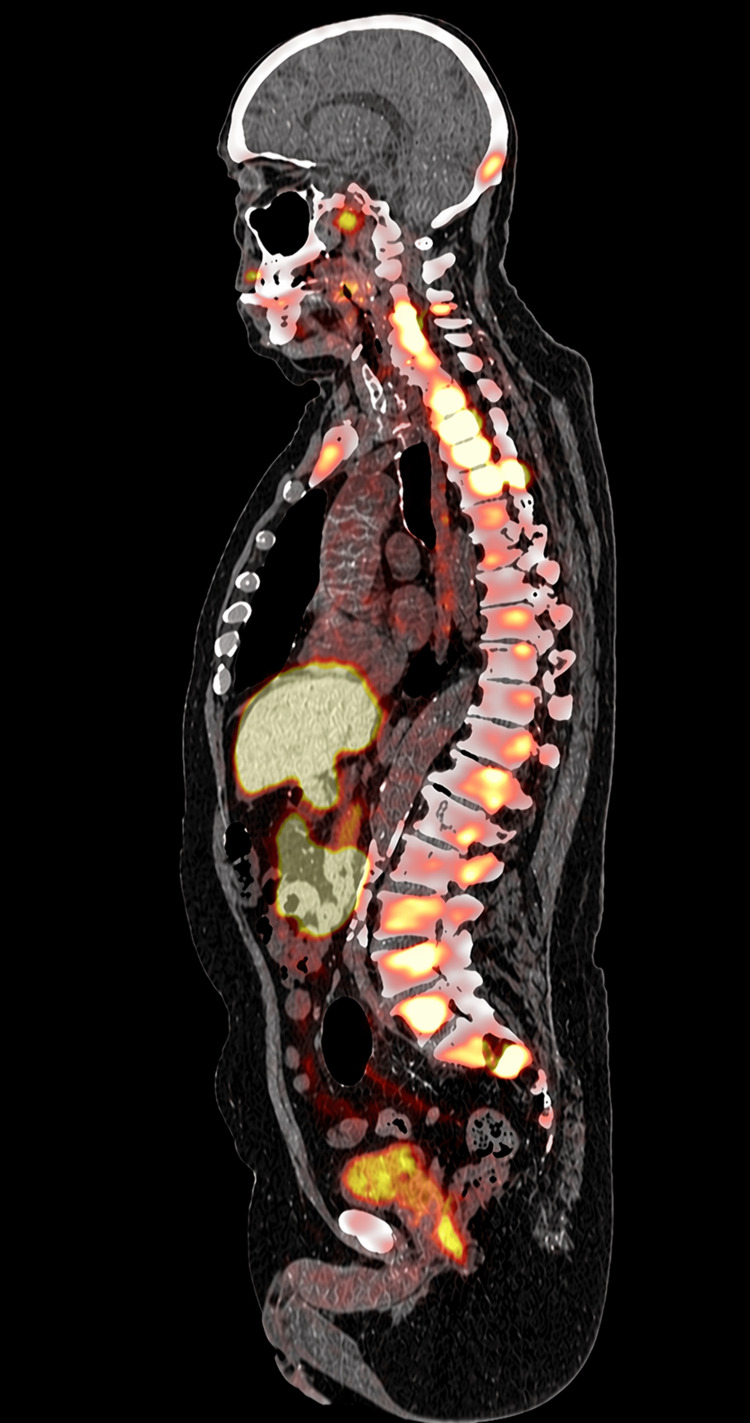
PSMA PET/CT (2025) showing partial response of metastatic disease. Follow-up imaging demonstrates decreased intensity of tracer uptake within previously involved skeletal sites, indicating partial metabolic response, with persistent residual disease.

Follow-up hybrid imaging with SPECT/CT in January 2026 confirmed a partial metabolic response of bone lesions. However, despite these imaging findings, PSA levels increased significantly, reaching 69.9 ng/mL in February 2026 (reference range: <4 ng/mL) (Figure 7).

**Figure 7:**
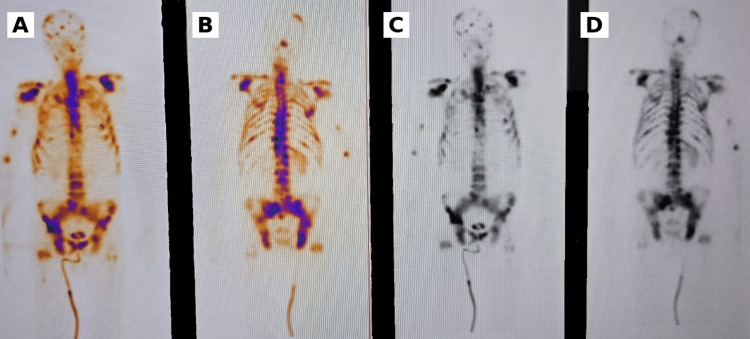
Bone scintigraphy showing diffuse metastatic skeletal disease. Whole-body images demonstrate heterogeneous, increased radiotracer uptake involving both axial and appendicular skeleton, consistent with extensive metastatic involvement. (A) Anterior whole-body planar image in color scale showing diffuse increased tracer uptake in the axial skeleton and pelvis. (B) Posterior whole-body planar image in color scale confirming widespread osseous metastatic disease. (C) Anterior grayscale image highlighting multiple metastatic lesions involving the spine, ribs, and pelvic bones. (D) Posterior grayscale image demonstrating persistent diffuse skeletal involvement consistent with advanced metastatic prostate cancer.

CT of the thorax and abdomen performed in February 2026 revealed extensive generalized bone metastases with a mixed pattern, including both osteosclerotic and osteolytic lesions, without evidence of visceral metastases or significant lymphadenopathy. Figure 8 illustrates the discordance between persistent thoracic radiologic findings and continuous biochemical progression observed during follow-up.

**Figure 8:**
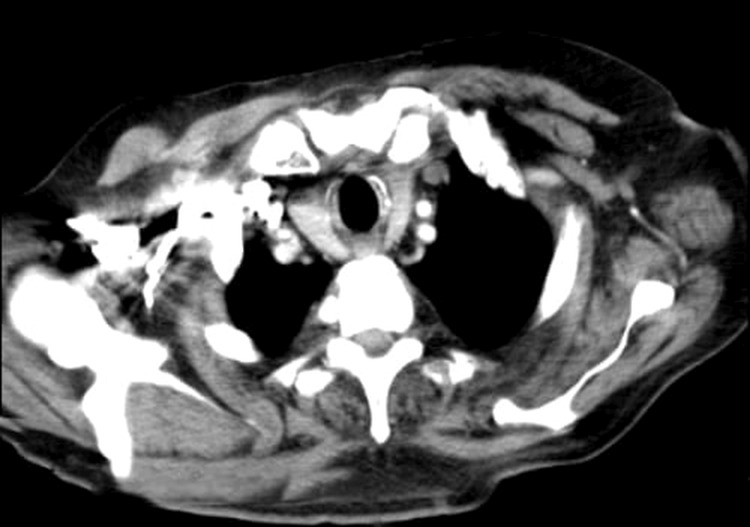
Representative thoracic CT image from February 2026 Axial thoracic CT image obtained during follow-up evaluation in February 2026, demonstrating persistent thoracic disease findings during systemic treatment monitoring.

Laboratory evaluation at that time demonstrated hemoglobin of 117 g/L (reference range: 120-160 g/L), white blood cell count of 4.84×10⁹/L (reference range: 4.0-10.0×10⁹/L), neutrophils of 2.68×10⁹/L (reference range: 1.8-7.4×10⁹/L), and lymphocytes of 1.26×10⁹/L (reference range: 0.8-4.3×10⁹/L), corresponding to a NLR of approximately 2.13. Erythrocyte sedimentation rate (ESR) was markedly elevated at 103 mm/h (reference range: <20 mm/h), while PSA measured 69.968 ng/mL (reference range: <4 ng/mL).

Serial laboratory assessment demonstrated progressive PSA elevation over time, accompanied by moderate temporal variation in inflammatory parameters, including NLR, despite relative radiologic disease control observed on follow-up imaging (Table 1).

**Table 1:** Longitudinal laboratory parameters during biochemical progression despite radiologic response NLR, neutrophil-to-lymphocyte ratio; PSA, prostate-specific antigen.

Parameter	Reference range	Unit	Feb 2022	Feb 2023	Feb 2024	Feb 2025	Feb 2026
Hemoglobin	120-160	g/L	135	102	133	111	117
Granulocytes	1.8-7.4	×10⁹/L	1.8	2.96	3.9	6.9	2.68
Lymphocytes	0.8-4.3	×10⁹/L	0.7	1.44	1.7	1.24	1.26
NLR	—	—	2.57	2.06	2.29	5.56	2.13
PSA	0-4.0	ng/mL	0	4.23	28.824	99.206	—

Despite relatively preserved functional status (ECOG 1), the patient demonstrated discordant disease dynamics, with follow-up imaging suggesting partial radiologic response, while biochemical progression persisted.

Denosumab (Xgeva) was discontinued following clinical suspicion of osteonecrosis after prolonged treatment exposure, and the patient was referred for further evaluation and consideration of second-line systemic therapy.

The observed discordance between radiologic/metabolic findings and biochemical progression may reflect ongoing systemic tumor activity not fully captured by imaging modalities alone. The coexistence of osteosclerotic and osteolytic lesions further suggests underlying biological heterogeneity, potentially reflecting variable therapeutic sensitivity across different metastatic compartments.

Longitudinal laboratory assessment demonstrated temporal variation in inflammatory parameters during the course of disease progression. Although some fluctuations in NLR appeared to occur alongside PSA changes, these findings remained modest and should be interpreted cautiously within the limitations of a single retrospective case observation.

Taken together, the clinical course illustrates the complexity of interpreting metastatic prostate cancer progression using isolated monitoring modalities. This case highlights the potential value of integrated longitudinal assessment incorporating imaging, biochemical, and inflammatory parameters during systemic treatment monitoring.

Further investigation in larger prospective cohorts is required to determine the clinical significance and reproducibility of these observations.

## Discussion

This case illustrates a clinically relevant discordance between radiologic response and biochemical progression in metastatic prostate cancer. Despite partial metabolic and radiologic response demonstrated on advanced imaging modalities, the patient exhibited persistent PSA elevation accompanied by inflammatory laboratory abnormalities. This dissociation highlights the complexity of disease monitoring in advanced prostate cancer and underscores the limitations of relying on a single modality for response assessment [6].

PSA remains a widely used surrogate marker of tumor burden and therapeutic response; however, its interpretation may become challenging in the setting of evolving treatment strategies and heterogeneous disease biology [7]. Although modern imaging modalities, including PSMA PET/CT, provide improved sensitivity for detecting metastatic lesions, they may not fully reflect the biological complexity of metastatic disease [8,9]. As demonstrated in this case, radiologic improvement did not fully correspond to biochemical disease control.

A plausible explanation for this discordant pattern is intratumoral heterogeneity and clonal evolution. Metastatic prostate cancer is increasingly recognized as a biologically heterogeneous disease composed of tumor subpopulations with variable treatment sensitivity [10,11]. The coexistence of osteosclerotic and osteolytic lesions in this patient may support the presence of such biological diversity across metastatic compartments.

An additional possibility involves tumor subclones with reduced or absent PSMA expression. Certain aggressive or dedifferentiated prostate cancer phenotypes, including neuroendocrine differentiation, may demonstrate reduced PSMA expression while retaining biological activity and PSA production. Consequently, some biologically active disease components may not be fully captured by PSMA-based imaging alone.

The switch from leuprorelin to goserelin was not expected to significantly influence disease trajectory, as both agents are luteinizing hormone-releasing hormone agonists with similar mechanisms of action. This modification was primarily related to logistical and treatment availability considerations and is unlikely to explain the observed biochemical progression. Denosumab was discontinued following clinical suspicion of osteonecrosis after prolonged exposure, although this event was not considered directly related to PSA kinetics.

Systemic inflammatory markers, including NLR, have been associated with prognosis and disease progression in several malignancies, including prostate cancer [12]. In the present case, inflammatory findings remained modest overall, particularly regarding NLR values, although ESR was markedly elevated. While temporal variation in inflammatory parameters was observed during disease progression, these findings should be interpreted cautiously within the limitations of a single retrospective case observation.

Importantly, this case emphasizes the potential value of integrated multimodal disease monitoring combining imaging, biochemical markers, and inflammatory parameters. Reliance solely on radiologic findings may underestimate ongoing disease activity in selected patients with heterogeneous metastatic burden [13].

Taken together, these observations highlight the complexity of interpreting disease progression in metastatic prostate cancer and support the need for further investigation into integrated longitudinal monitoring strategies.

Limitations

This report has several limitations. First, it represents a single clinical case, limiting the generalizability of the observations. Second, the proposed role of systemic inflammatory markers, including the NLR, as early indicators of disease progression remains hypothesis-generating, as the data are retrospective and correlative rather than causal. Third, temporal correlation between radiologic and biochemical findings may be limited by the retrospective nature of imaging review and the availability of follow-up imaging documentation. Furthermore, no repeat biopsy or molecular profiling was available to directly confirm clonal evolution, PSMA-negative subclones, or neuroendocrine differentiation, and these interpretations remain biologically plausible but speculative. In addition, no formal statistical analysis was performed, consistent with the nature of a case report.

Finally, although temporal variation in inflammatory markers was observed during the disease course, these findings remained modest and non-specific and may have been influenced by multiple factors, including tumor biology, systemic inflammatory status, treatment effects, or intercurrent clinical conditions. Consequently, the observed associations should be interpreted cautiously and considered hypothesis-generating rather than definitive evidence of causal biological relationships.

## Conclusions

This case highlights the limitations of relying solely on imaging-based criteria in the assessment of treatment response in metastatic prostate cancer. Discordance between radiologic findings and biochemical progression may reflect underlying tumor heterogeneity, clonal evolution, and asynchronous treatment response across different metastatic compartments. In the present case, temporal variation in inflammatory markers was observed alongside biochemical progression, although these findings remained modest and should be interpreted cautiously within the limitations of a single retrospective case report.

These observations support the potential value of an integrated multimodal approach to disease monitoring that incorporates imaging, biochemical markers such as PSA, and selected inflammatory parameters. Further studies are warranted to better characterize discordant response patterns and to determine the clinical relevance of combined longitudinal monitoring strategies in metastatic prostate cancer.
